# *NINJ1* Defines a Hyperinflammatory Monocyte State Associated with Severe COVID-19

**DOI:** 10.3390/biomedicines14102203

**Published:** 2026-09-29

**Authors:** Fang Yu, Hao Ma, Muyuan Li, Huali Zhang, Yiying Yang

**Affiliations:** 1 Department of Rheumatology and Immunology, Xiangya Hospital, Department of Pathophysiology, Xiangya School of Basic Medicine Science, Central South University, Changsha 410000, China; 2 Sepsis Translational Medicine Key Lab of Hunan Province, Changsha 410000, China; 3 Postdoctoral Research Station of Biology, Xiangya School of Basic Medicine Science, Central South University, Changsha 410000, China; 4 National Clinical Research Center for Geriatric Disorders, Xiangya Hospital, Central South University, Changsha 410000, China

**Keywords:** COVID-19, NINJ1, inflammatory monocytes, single-cell RNA sequencing, immune dysregulation, prognostic biomarker

## Abstract

**Background/Objectives**: Severe coronavirus disease 2019 (COVID-19) is characterized by dysregulated immune activation and excessive inflammation; however, the specific immune cell states that drive disease progression remain incompletely understood. **Methods**: Here, we integrated single-cell RNA sequencing analysis of peripheral blood mononuclear cells with clinical validation to investigate the role of Ninjurin-1 (NINJ1) in COVID-19-associated immune dysregulation. **Results**: Single-cell profiling revealed that NINJ1 expression was largely confined to monocytes and defined as a distinct *NINJ1*^+^ inflammatory monocyte state in patients with COVID-19. Compared with NINJ1^−^ monocytes, *NINJ1*^+^ monocytes exhibited enhanced inflammatory transcriptional programs, altered regulon activity, and increased cell–cell communication capacity, suggesting their potential role as a highly activated immune state involved in inflammatory amplification. Further ligand–receptor interaction analysis demonstrated that *NINJ1*^+^ monocytes functioned as key communication hubs within the COVID-19 immune landscape, coordinating extensive interactions with other immune populations. In an independent cohort of 99 COVID-19 patients and 51 healthy controls, serum NINJ1 levels were significantly elevated in infected individuals and were further increased in non-survivors. Circulating NINJ1 levels correlated with inflammatory and immune dysregulation indicators, including C-reactive protein, procalcitonin, prothrombin time, neutrophil-to-lymphocyte ratio, and lymphocyte depletion. Receiver operating characteristic analysis demonstrated that serum NINJ1 exhibited significant predictive value for in-hospital mortality. **Conclusions**: Collectively, our findings identify *NINJ1*^+^ inflammatory monocytes as an activated immune state associated with the transition from protective innate defense to pathological inflammation, providing new insights into COVID-19 immunopathogenesis and highlighting NINJ1 as a promising biomarker candidate for risk stratification and therapeutic exploration.

## 1. Introduction

Coronavirus disease 2019 (COVID-19), caused by infection with severe acute respiratory syndrome coronavirus 2 (SARS-CoV-2), has resulted in substantial global morbidity and mortality [1,2,3,4,5]. Although most individuals experience mild or moderate disease, a subset of patients develop severe manifestations characterized by excessive inflammation, immune dysregulation, coagulation abnormalities, and multiorgan dysfunction [6,7,8,9,10,11,12]. Increasing evidence indicates that clinical deterioration in COVID-19 is not solely determined by viral replication, but is largely driven by maladaptive host immune responses [13,14,15,16]. However, the cellular and molecular mechanisms underlying pathogenic immune activation and the identification of reliable predictors of adverse outcomes remain incompletely understood.

Single-cell RNA sequencing (scRNA-seq) studies have substantially advanced our understanding of immune heterogeneity during SARS-CoV-2 infection, revealing profound alterations in circulating immune cell composition and transcriptional states [17,18,19,20,21,22]. Among these changes, monocytes have emerged as critical regulators of COVID-19-associated inflammation [17,23,24,25,26,27,28]. Distinct monocyte subsets characterized by enhanced interferon signaling, inflammatory cytokine production, and antigen presentation have been associated with disease severity. However, monocytes represent a highly heterogeneous population, and the specific monocyte states that contribute to uncontrolled inflammation and poor clinical outcomes remain to be fully defined.

Ninjurin-1 (NINJ1) is a highly conserved cell adhesion molecule involved in inflammatory responses, immune cell migration, and tissue injury [29,30,31,32,33,34]. Recent studies have implicated NINJ1 as an important regulator of inflammatory cell death and innate immune activation, suggesting that it may serve as a molecular link between cellular stress and inflammatory amplification. Nevertheless, the expression pattern, cellular source, and clinical significance of NINJ1 during viral infection, particularly in COVID-19, remain largely unknown.

In this study, we integrated single-cell transcriptomic analysis of peripheral blood mononuclear cells with clinical validation in an independent COVID-19 cohort to investigate the role of NINJ1 in SARS-CoV-2-induced immune dysregulation. We identified a distinct *NINJ1*^+^ inflammatory monocyte state in patients with COVID-19, characterized by enhanced inflammatory transcriptional programs, altered regulon activity, and extensive cell–cell communication networks. Furthermore, we demonstrated that circulating NINJ1 levels were elevated in patients with COVID-19 and were associated with inflammatory markers, disease severity, and in-hospital mortality. These findings identify NINJ1 as a marker of an activated monocyte state associated with adverse clinical outcomes and highlight its potential utility as a prognostic biomarker for adverse outcomes in COVID-19.

## 2. Methods and Materials

### 2.1. Single-Cell RNA Sequencing Data Processing and Cell Annotation

Publicly available peripheral blood mononuclear cells (PBMCs) scRNA-seq data were obtained from the Gene Expression Omnibus database (GSE149689) [35,36], from which samples from patients with COVID-19 and healthy controls were selected for the present analysis. Cells with low-quality features, including fewer than 200 detected genes, fewer than 1000 or more than 15,000 unique molecular identifiers (UMIs), or mitochondrial transcript proportion >15%, were excluded. Doublets were identified and removed using scDblFinder. Data normalization, dimensionality reduction, clustering, and batch correction were performed using Seurat and Harmony. Cell types were annotated based on canonical marker genes from the AIFI Immune Health Atlas. Major immune populations were classified into T cells, B cells, NK cells, monocytes, and dendritic cells (DCs). Monocytes were subsequently extracted and re-clustered into seven subsets, including IL1β^+^ CD14, ISG^+^ CD14, Core CD14, C1Q^+^ CD16, ISG^+^ CD16, Core CD16, and intermediate monocytes. *NINJ1* expression was evaluated based on both average expression levels and the proportion of *NINJ1*-expressing cells. Values shown in the figures were calculated across all cells pooled within each group, whereas between-group comparisons of cell-type composition and NINJ1 expression were performed at the donor level, using the Wilcoxon rank-sum test with Benjamini–Hochberg correction, because cells from the same donor are not statistically independent.

### 2.2. Differential Expression and Functional Enrichment Analysis

Monocytes were classified as *NINJ1*^+^ or *NINJ1*^−^ according to detectable or undetectable NINJ1 transcript expression. Differentially expressed genes (DEGs) between *NINJ1*^+^ and *NINJ1*^−^ monocytes were identified using the Wilcoxon rank-sum test implemented in Seurat. Genes with adjusted *p* < 0.05 and |log_2_ fold change| > 0.5 were considered significant.

Functional enrichment analyses, including Gene Ontology (GO) and Kyoto Encyclopedia of Genes and Genomes (KEGG) pathway analyses, were performed using clusterProfiler. Multiple testing correction was performed using the Benjamini–Hochberg method. Enrichment analyses were conducted separately for genes upregulated and downregulated in *NINJ1*^+^ monocytes.

### 2.3. Single-Cell Regulon Activity Analysis

Transcription factor regulatory activity was inferred using the SCENIC workflow [37]. Gene regulatory networks were constructed using GENIE3; transcription factor–target modules were refined using RcisTarget; and regulon activity scores were calculated using AUCell. This analysis was restricted to monocytes from the COVID-19 group, and 1489 *NINJ1*^+^ and 1489 *NINJ1*^−^ monocytes were randomly selected by downsampling so that the two populations were equally represented. The correspondence between the original sample identifiers and the new donor labels, together with the number of *NINJ1*^+^ and *NINJ1*^−^ monocytes contributed by each donor, is now provided in Supplementary Table S3.

Regulon activity scores were then compared between the two populations. Differential regulon activity between *NINJ1*^+^ and *NINJ1*^−^ monocytes was assessed using the Wilcoxon rank-sum test followed by Benjamini–Hochberg correction for multiple testing, and ΔAUCell was calculated as the difference in mean AUCell scores between *NINJ1*^+^ and *NINJ1*^−^ monocytes. Regulons were classified as activated or suppressed when the adjusted *p* value was <0.05 and the direction of change was consistent in at least 9 of the 11 donors, and as not significant otherwise.

### 2.4. Cell–Cell Communication Analysis

Cell–cell communication networks were analyzed using CellChat based on ligand–receptor interactions [38]. Analyses were performed separately in the COVID-19 and healthy control groups. Immune populations, including major lymphocyte populations and refined monocyte subsets stratified by NINJ1 expression, were defined as distinct cell populations. Within each group, significant ligand–receptor interactions were identified by permutation testing (20 permutations, *p* < 0.05). Differences in interaction number, interaction strength, and signaling activity between the two groups were then compared descriptively, without formal statistical testing. Signaling pathways originating from *NINJ1*^+^ monocytes were further characterized and visualized using chord diagrams.

### 2.5. Study Design and Participants

This retrospective cohort study included 99 patients with COVID-19 admitted to Xiangya Hospital, Central South University, between January and February 2023, together with 51 healthy controls. Patients were included if they were ≥18 years old and had laboratory-confirmed SARS-CoV-2 infection. Patients with incomplete clinical information or non-COVID-19-related conditions affecting inflammatory assessment were excluded. The study was approved by the Ethics Committee of Xiangya Hospital, Central South University (No. 201212074), and conducted in accordance with the Declaration of Helsinki.

### 2.6. Diagnosis and Clinical Outcomes

COVID-19 diagnosis was established based on clinical manifestations and laboratory confirmation of SARS-CoV-2 infection by reverse transcription polymerase chain reaction (RT-PCR) from nasopharyngeal swab samples. The primary outcome was in-hospital mortality. Patients discharged alive were classified as survivors, whereas patients who died during hospitalization were classified as non-survivors.

### 2.7. Serum NINJ1 Measurement

Blood samples were collected within 24 h after COVID-19 diagnosis. Serum samples were obtained by centrifugation and stored at −80 °C until analysis. Serum NINJ1 concentrations were measured using a commercially available human NINJ1 ELISA kit (CUSABIO, CSB-EL015808HU) according to the manufacturer’s instructions.

### 2.8. Statistical Analysis

Continuous variables were presented as medians and interquartile ranges (IQRs) and compared using the Mann–Whitney U test. Categorical variables were expressed as numbers and percentages. Correlations between serum NINJ1 levels and clinical laboratory parameters were assessed using Spearman correlation analysis. The predictive value of serum NINJ1 for in-hospital mortality was evaluated using receiver operating characteristic (ROC) curve analysis, including calculation of the area under the curve (AUC), sensitivity, specificity, and optimal cutoff value. Statistical analyses were performed using SPSS 27.0 and GraphPad Prism 10.0. A two-sided *p* value <0.05 was considered statistically significant.

## 3. Results

### 3.1. Single-Cell Profiling Reveals NINJ1-Associated Inflammatory Monocyte States in COVID-19

To characterize the immune landscape associated with COVID-19 and identify cell populations potentially involved in disease progression, we performed scRNA-seq analysis of PBMCs from patients with COVID-19 and healthy controls. After quality control and dimensionality reduction, 37,315 high-quality cells (22,456 from 11 patients with COVID-19 and 14,859 from 4 healthy controls) were retained, and major immune populations were identified based on canonical marker expression, including T cells, B cells, NK cells, monocytes, and DCs (Figure 1A–C).

Comparison of immune cell composition showed a trend towards a higher monocyte and B cell proportion and a lower NK cell proportion in the COVID-19 group (Figure 1B). However, when proportions were recalculated per donor, none of these differences reached statistical significance after multiple-testing correction (all FDR > 0.05), which likely reflects the limited number of donors and the substantial inter-individual variation in this dataset. Given the emerging evidence implicating inflammatory monocytes in COVID-19-associated immune dysregulation, we further interrogated monocyte heterogeneity. Unsupervised clustering resolved seven transcriptionally distinct monocyte subsets, including IL1β^+^ CD14 monocytes, ISG^+^ CD14 monocytes, Core CD14 monocytes, C1Q^+^ CD16 monocytes, ISG^+^ CD16 monocytes, Core CD16 monocytes, and intermediate monocytes, which were distinguished by their characteristic marker gene expression patterns (Figure 1E,F). The monocyte compartment showed a pronounced compositional shift in the COVID-19 group. Among all monocytes analyzed, IL1β^+^ CD14 monocytes accounted for 35.9% in the COVID-19 group versus 2.6% in the healthy control group (median per-donor proportion 22.0% versus 2.8%), whereas Core CD16 and ISG^+^ CD16 monocytes were markedly less frequent in the COVID-19 group (3.9% versus 14.1% and 0.3% versus 1.3%, respectively). The direction of these changes was consistent across most individual donors (uncorrected *p* = 0.014–0.037 for Core CD16, ISG^+^ CD16 and IL1β^+^ CD14), but none of the seven subsets reached statistical significance after correction for multiple testing (smallest FDR = 0.085). 

Next, we examined the cellular distribution of NINJ1, a membrane-associated inflammatory mediator implicated in immune activation. NINJ1 expression was largely confined to monocytes, in which the median per-donor proportion of NINJ1^+^ cells reached 82.3% in the COVID-19 group, compared with 11.8–27.6% across the lymphoid lineages and DCs (Figure 1D). Within monocytes, however, neither the proportion of NINJ1^+^ cells nor the mean NINJ1 expression differed significantly between the two groups at the donor level (FDR = 0.84 and 0.63, respectively), indicating that NINJ1 demarcates a monocyte-restricted state rather than a transcript induced by infection per se. Consistent with this, NINJ1^+^ cells were present in across all seven monocyte subsets in both groups (Figure 1G). These findings identify NINJ1 as a monocyte-associated inflammatory signature and suggest its potential involvement in COVID-19-associated immune perturbation.

### 3.2. NINJ1^+^ Monocytes Exhibit Enhanced Inflammatory Transcriptional Programs and Regulatory Activation

To define the molecular characteristics of *NINJ1*-expressing monocytes, we performed differential transcriptomic analysis between *NINJ1*^+^ and *NINJ1*^−^ monocytes isolated from COVID-19 patients. Volcano plot analysis demonstrated extensive transcriptional differences between the two populations, with *NINJ1*^+^ monocytes displaying a distinct gene expression signature (Figure 2A). A heatmap analysis of the top differentially expressed genes further confirmed that *NINJ1*^+^ monocytes from COVID-19 patients exhibited a unique transcriptional profile compared with *NINJ1*^−^ monocytes, while this signature was less prominent in monocytes from healthy controls (Figure 2B). These findings indicate that *NINJ1* expression identifies a disease-associated monocyte state rather than representing a constitutive monocyte marker. To gain insight into the biological functions associated with *NINJ1*^+^ monocytes, we performed functional enrichment analysis of genes upregulated in *NINJ1*^+^ cells. GO analysis revealed significant enrichment of pathways involved in inflammatory responses, innate immune activation, cytokine production, and immune effector functions (Figure 2C). Consistently, KEGG pathway analysis demonstrated activation of inflammation-related signaling pathways, including pathways associated with host defense and immune regulation (Figure 2D). Because transcriptional programs are governed by coordinated activity of transcription factors, we further performed single-cell regulon activity analysis. To identify the transcription factors underlying this phenotype, we inferred single-cell regulon activity within the COVID-19 group, so that NINJ1 status would not be confounded by disease status. NINJ1^+^ monocytes displayed higher activity of the NF-κB (NFKB2), AP-1 (JUND, FOSB) and interferon-regulated (STAT1, IRF1, IRF7) regulons, together with SPI1, POU2F2, RUNX3 and KLF2, whereas regulons associated with progenitor and homeostatic programs (MEIS1, PBX1, TAL1, ELF1, IRF8) were lower compared with NINJ1^−^ monocytes (Figure 2E). Of the regulons examined, 19 were classified as activated and 31 as suppressed, indicating broad remodeling of transcriptional regulatory networks. Collectively, these results demonstrate that *NINJ1* marks a transcriptionally activated monocyte population characterized by heightened inflammatory signaling and distinct regulatory states during COVID-19.

### 3.3. NINJ1^+^ Monocytes Function as Inflammatory Communication Hubs During COVID-19

Given the central role of monocytes in coordinating immune interactions, we next investigated whether *NINJ1*^+^ monocytes contribute to altered intercellular communication during COVID-19. Cell–cell communication analysis revealed extensive remodeling of immune interaction networks in COVID-19 patients compared with healthy controls, with substantial changes in both interaction numbers and communication strength (Figure 3A). Analysis of signaling roles across immune populations demonstrated that monocytes acquired prominent sender characteristics in COVID-19, exhibiting increased outgoing signaling activity compared with controls (Figure 3B). Detailed analysis of incoming and outgoing signaling patterns within the COVID-19 group further revealed that the ANNEXIN, GALECTIN, MIF and RESISTIN pathways accounted for the largest share of signaling activity, with NINJ1^+^ monocyte subsets among the most active senders (Figure 3C). To specifically determine the contribution of *NINJ1*^+^ monocytes, we analyzed ligand–receptor communication pathways originating from this population. *NINJ1*^+^ monocytes exhibited extensive interactions with multiple immune cell subsets through diverse signaling pathways, forming a highly connected inflammatory communication network (Figure 3D). These interactions involved both monocyte-derived signaling toward immune effector cells and reciprocal regulatory signals from surrounding populations.

Together, these findings suggest that *NINJ1*^+^ monocytes represent a highly interactive inflammatory hub that may amplify immune dysregulation through enhanced cell–cell communication during COVID-19.

### 3.4. Circulating NINJ1 Is Associated with Disease Severity and Mortality in COVID-19

To determine whether the *NINJ1*-associated inflammatory signature identified by single-cell transcriptomic analysis was reflected at the systemic protein level and associated with clinical outcomes, we quantified serum NINJ1 concentrations in an independent cohort consisting of 99 patients with COVID-19, including 80 survivors and 19 non-survivors, and 51 healthy controls. Detailed clinical characteristics and laboratory measurements are summarized in Supplementary Tables S1 and S2.

Serum NINJ1 levels were significantly elevated in patients with COVID-19 compared with healthy controls (Figure 4A, *p* < 0.01). Within the COVID-19 cohort, patients who died during hospitalization exhibited markedly higher circulating NINJ1 concentrations than survivors (Figure 4B, *p* < 0.001). These findings demonstrate that increased circulating NINJ1 is associated with COVID-19 and is further associated with adverse clinical outcomes, supporting its potential relevance to disease severity and mortality.

To further investigate the clinical relevance of circulating NINJ1, we examined correlation analyses between admission serum NINJ1 levels and routine laboratory parameters associated with COVID-19 progression (Figure 4C). Serum NINJ1 concentrations showed significant positive correlations with inflammatory markers, including C-reactive protein (CRP) and procalcitonin (PCT), as well as coagulation-related and hematological indicators, including prothrombin time (PT), white blood cell counts, neutrophil counts, and neutrophil-to-lymphocyte ratio (NLR). Conversely, NINJ1 levels were inversely correlated with lymphocyte counts. These associations indicate that elevated circulating NINJ1 is closely linked to systemic inflammation, immune imbalance, and coagulation abnormalities characteristic of severe COVID-19.

We next evaluated the ability of serum NINJ1 for in-hospital mortality using ROC curve analysis. Serum NINJ1 showed significant discriminatory performance for in-hospital mortality, with an AUC of 0.7934 (*p* < 0.0001) (Figure 4D, Table 1). Among the evaluated biomarkers, NLR demonstrated the highest discriminatory performance (AUC = 0.8794, *p* < 0.0001), whereas NINJ1 showed higher AUC values than CRP (AUC = 0.7323, *p* = 0.0053), D-dimer (AUC = 0.7093, *p* = 0.0071), and IL-6 (AUC = 0.6930, *p* = 0.0111). Thus, serum NINJ1 demonstrated potential value for identifying patients at increased risk of in-hospital mortality and may provide complementary information to established inflammatory indicators. However, these findings do not establish NINJ1 as an independent prognostic factor, and further studies incorporating multivariable adjustment and larger prospective cohorts are required to determine its independent prognostic value. 

## 4. Discussion

The clinical manifestations of COVID-19 are highly heterogeneous, ranging from asymptomatic infection to life-threatening respiratory failure and systemic inflammatory complications. Although advances in antiviral therapies and supportive care have substantially improved patient outcomes, severe COVID-19 remains associated with excessive immune activation, dysregulated inflammation, and immune-mediated tissue injury. Increasing evidence indicates that disease severity is largely determined by the host immune response rather than viral replication alone. However, the specific immune cell states that initiate and sustain pathological inflammation remain incompletely characterized.

In this study, by integrating single-cell transcriptomic profiling with clinical validation, we identified *NINJ1*^+^ inflammatory monocytes as a previously unrecognized immune state associated with COVID-19 progression and poor clinical outcomes. Our findings demonstrate that NINJ1 expression is preferentially enriched in inflammatory monocyte populations during SARS-CoV-2 infection and is associated with enhanced inflammatory transcriptional programs, altered transcription factor activity, and extensive intercellular communication networks. Importantly, elevated circulating NINJ1 levels were confirmed in an independent patient cohort and were associated with inflammatory burden and in-hospital mortality. Collectively, these findings establish NINJ1 as a potential molecular link between monocyte activation and adverse clinical outcomes in COVID-19.

NINJ1 has traditionally been recognized as a cell adhesion molecule involved in leukocyte migration and inflammatory responses [39,40,41,42,43,44,45]. Recent studies have further implicated NINJ1 in regulating inflammatory amplification and tissue injury responses, suggesting that NINJ1 may act as an important modulator of innate immune activation [46,47,48]. However, whether NINJ1 contributes to immune dysregulation during viral infection remains largely unknown. In this study, we identified *NINJ1*^+^ monocytes as a distinct inflammatory immune state that appears to be positioned at the interface between protective innate defense and pathological inflammation during COVID-19.

Unlike conventional inflammatory markers that simply reflect immune activation, NINJ1 expression identified a transcriptionally and functionally specialized monocyte population characterized by enhanced inflammatory programs, altered transcriptional regulatory activity, and extensive intercellular communication capacity [49,50]. These findings suggest that *NINJ1*^+^ monocytes may occupy a critical position in the transition from an effective antiviral response to excessive and potentially harmful inflammation. Under physiological conditions, monocyte activation is essential for pathogen clearance and immune defense; however, persistent activation or inappropriate amplification may contribute to cytokine dysregulation, immune-mediated tissue damage, and organ dysfunction. Therefore, *NINJ1*^+^ monocytes may represent a cellular state in which protective innate immunity becomes uncoupled from inflammatory control, thereby contributing to disease progression.

Our cell–cell communication analysis further supports this concept by demonstrating that *NINJ1*^+^ monocytes function as highly connected inflammatory hubs within the COVID-19 immune landscape. These cells exhibited enhanced outgoing signaling activity and extensive ligand–receptor interactions with diverse immune populations, suggesting that they may coordinate inflammatory communication across the immune system. Rather than acting as isolated responders, *NINJ1*^+^ monocytes appear to participate in a broader immune regulatory network that determines whether antiviral immunity resolves or progresses toward pathological inflammation. This concept provides a potential mechanistic explanation for why excessive monocyte activation is frequently associated with severe COVID-19.

An important translational implication of our study is the identification of circulating NINJ1 as a potential prognostic biomarker. Serum NINJ1 levels were significantly increased in COVID-19 patients and were further elevated among non-survivors. Furthermore, circulating NINJ1 correlated positively with established indicators of systemic inflammation, including CRP, PCT, and NLR, while showing an inverse association with lymphocyte counts, consistent with the immunological features of severe COVID-19. ROC analysis demonstrated that NINJ1 exhibited significant predictive performance for in-hospital mortality and outperformed several commonly used inflammatory biomarkers, including CRP, D-dimer, and IL-6. These findings suggest that NINJ1 may provide complementary prognostic information reflecting inflammatory immune activation. However, given that NLR showed higher predictive performance in our cohort, NINJ1 should be considered as a complementary biomarker rather than a replacement for existing clinical indicators.

The clinical relevance of NINJ1 may extend beyond biomarker potential. Given its emerging role in inflammatory amplification, NINJ1 may represent a candidate therapeutic target for modulating excessive immune activation. Targeting NINJ1-associated monocyte activation could potentially attenuate pathological inflammation while preserving essential antiviral immune responses. Future studies using genetic models or pharmacological inhibition approaches will be required to determine whether NINJ1 directly regulates monocyte inflammatory function and whether therapeutic intervention targeting the NINJ1 pathway can improve outcomes in severe viral infections.

Several limitations should be acknowledged. First, our study was based on retrospective clinical cohorts, and the relatively limited number of non-survivors (19 of 99 patients with COVID-19) restricted the statistical power for multivariable analyses. Therefore, we did not establish whether serum NINJ1 provides independent prognostic information after adjustment for age, comorbidities, and other established clinical risk factors. Larger prospective, multicenter cohorts with comprehensive clinical information are warranted to validate the prognostic relevance of circulating NINJ1. Second, although integrated single-cell analyses revealed a strong association between *NINJ1* expression and inflammatory monocyte activation, the observational nature of our study precludes conclusions regarding causality. Functional experiments are therefore needed to determine whether NINJ1 directly contributes to monocyte-mediated immune dysregulation or primarily marks an activated inflammatory state. Third, *NINJ1*^+^ and *NINJ1*^−^ monocytes were defined according to the presence or absence of detectable *NINJ1* transcripts, which may be affected by the dropout inherent to single-cell RNA sequencing; alternative expression thresholds and orthogonal validation will be important to confirm the robustness of this classification. Fourth, circulating NINJ1 levels were assessed at admission, and longitudinal analyses will be necessary to determine whether dynamic changes in NINJ1 correlate with disease progression, clinical outcomes, or therapeutic responses. Fifth, although CellChat analysis suggested extensive intercellular communication involving *NINJ1*^+^ monocytes, these ligand–receptor interactions were computationally inferred and require experimental validation. Finally, although our analyses identified *NINJ1*^+^ monocytes as a distinct inflammatory population, the upstream mechanisms driving NINJ1 induction during SARS-CoV-2 infection and the functional consequences of NINJ1 upregulation remain to be elucidated.

## 5. Conclusions

In conclusion, our study identifies *NINJ1*^+^ inflammatory monocytes as a highly activated inflammatory state associated with COVID-19 severity and mortality. By integrating single-cell immune profiling with clinical validation, we reveal a previously unrecognized role for NINJ1 in shaping inflammatory monocyte programs and immune communication networks. These findings provide new insights into COVID-19 immunopathogenesis by identifying *NINJ1*^+^ monocytes as a state positioned at the interface between protective innate defense and pathological inflammation. Beyond its potential utility as a prognostic biomarker, NINJ1 may represent a candidate target for modulating maladaptive immune activation while preserving antiviral immunity.

## Figures and Tables

**Figure 1:**
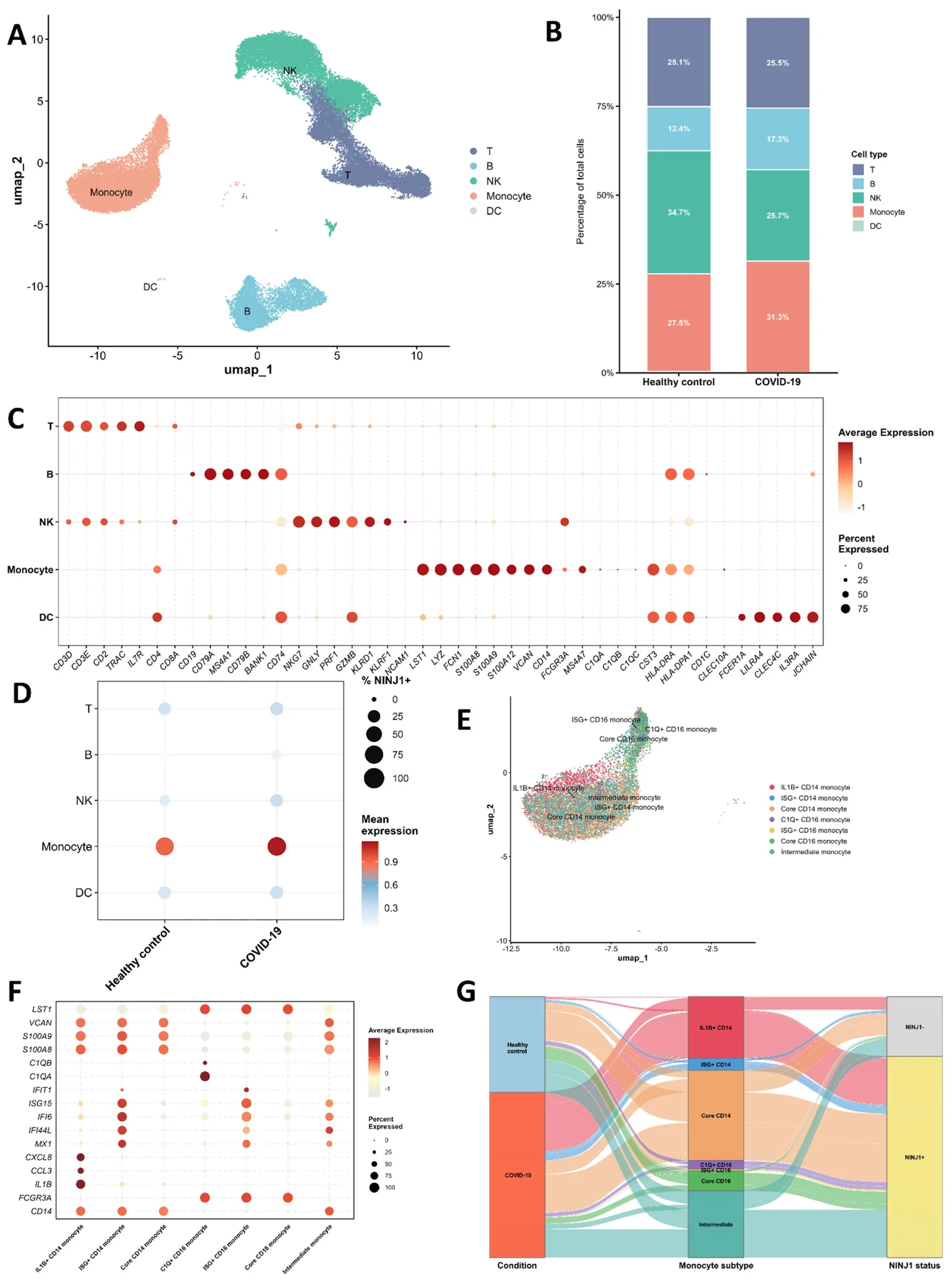
Single-cell transcriptomic profiling identifies NINJ1-associated inflammatory monocyte states in COVID-19. (**A**) Uniform manifold approximation and projection (UMAP) visualization of peripheral blood mononuclear cells (PBMCs) from patients with COVID-19 and healthy controls, showing major immune populations including T cells, B cells, natural killer (NK) cells, monocytes, and dendritic cells (DCs). (**B**) Relative abundance of major immune cell populations in COVID-19 patients and healthy controls. Bars show the proportion of each lineage among all cells pooled within each group. (**C**) Dot plot showing the expression patterns of canonical marker genes used for immune cell annotation. Dot size represents the proportion of cells expressing each marker gene, and color indicates scaled average expression. (**D**) NINJ1 expression level across major immune populations in the COVID-19 and healthy control groups. Dot size indicates the proportion of NINJ1-expressing cells, and color represents mean NINJ1 expression levels. (**E**) UMAP visualization of seven transcriptionally distinct monocyte subsets identified after monocyte re-clustering, including IL1B^+^ CD14, ISG^+^ CD14, Core CD14, C1Q^+^ CD16, ISG^+^ CD16, Core CD16, and intermediate monocytes. (**F**) Dot plot showing representative marker genes defining the seven monocyte subsets. Dot size and color represent the percentage of expressing cells and scaled average expression, respectively. (**G**) Sankey diagram illustrating the distribution of monocytes according to disease status, monocyte subtype, and NINJ1 expression status. NINJ1^+^ cells were defined based on detectable NINJ1 transcript expression, and band width represents the relative number of cells.

**Figure 2:**
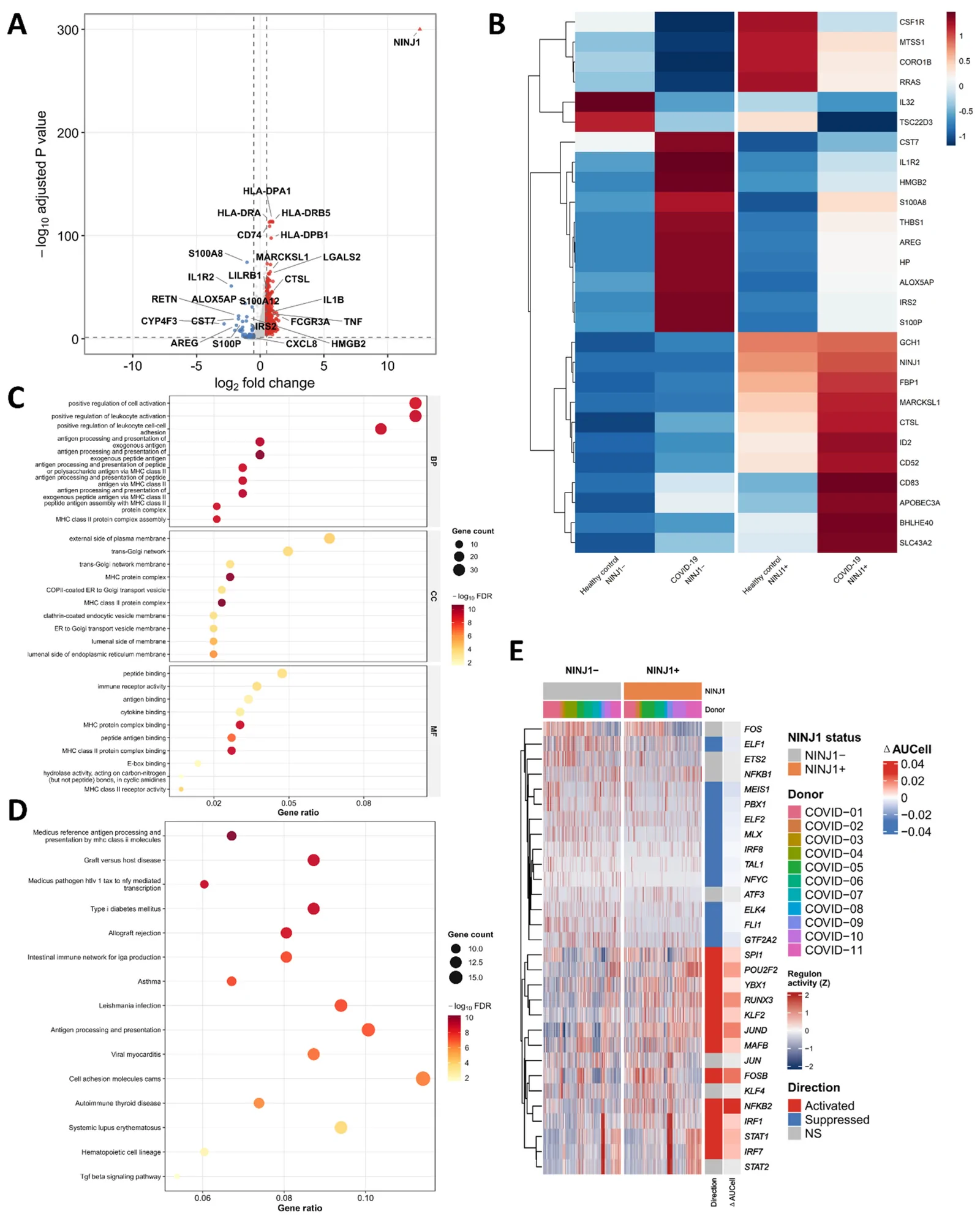
NINJ1^+^ monocytes exhibit enhanced inflammatory transcriptional programs and altered regulatory activity in COVID-19. (**A**) Volcano plot showing differentially expressed genes between NINJ1^+^ and NINJ1^−^ monocytes from patients with COVID-19. Genes significantly upregulated in NINJ1^+^ monocytes are shown in red, whereas genes downregulated in NINJ1^+^ monocytes are shown in blue. NINJ1 expression is highlighted. Differentially expressed genes were defined as adjusted *p* < 0.05 and |log_2_ fold change| > 0.5. (**B**) Heatmap displaying the top differentially expressed genes between NINJ1^+^ and NINJ1^−^ monocytes across COVID-19 and healthy control groups. Expression values represent row-scaled Z scores of average gene expression. (**C**) Gene Ontology (GO) enrichment analysis of genes upregulated in NINJ1^+^ monocytes from COVID-19 patients. Biological process, cellular component, and molecular function categories are shown. Dot size represents gene number, and color indicates statistical significance. (**D**) Kyoto Encyclopedia of Genes and Genomes (KEGG) pathway enrichment analysis of genes upregulated in NINJ1^+^ monocytes. Dot size indicates the number of enriched genes, and color represents adjusted significance values. (**E**) Single-cell regulon activity analysis comparing NINJ1^+^ and NINJ1^−^ monocytes from the COVID-19 group. Heatmap displays row-scaled regulon activity scores across individual cells, with donor identity indicated by annotation bars. ΔAUCell is the difference in mean AUCell regulon activity between NINJ1^+^ and NINJ1^−^ monocytes and represents the effect size on the original AUCell scale. Direction classifies each regulon as activated, suppressed, or not significant (NS).

**Figure 3:**
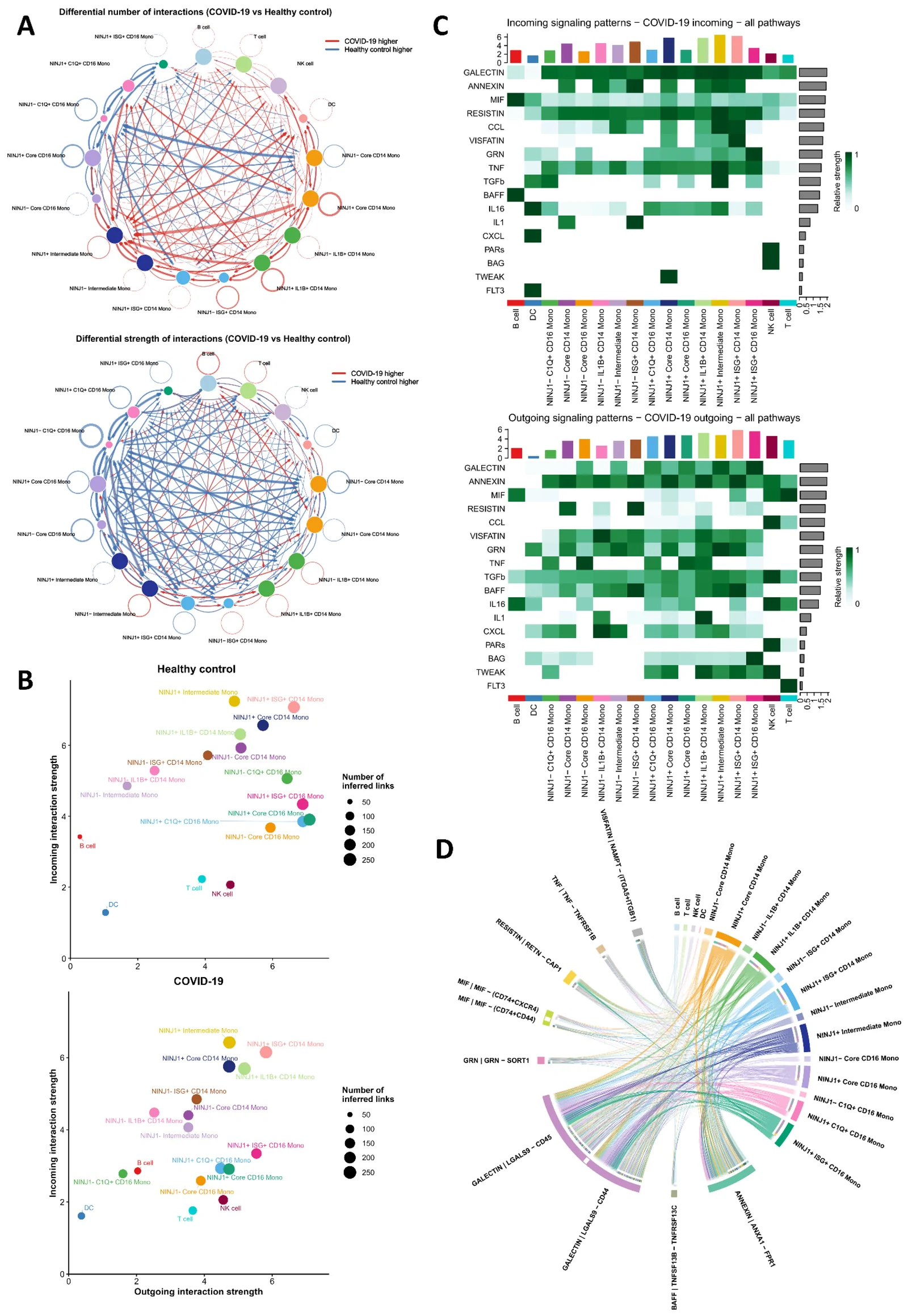
NINJ1^+^ monocytes reshape immune communication networks during COVID-19. (**A**) Differential cell–cell communication networks between COVID-19 patients and healthy controls inferred by ligand–receptor interactions. Networks are shown according to interaction number (upper panel) and interaction strength (lower panel). Red and blue edges represent interactions that increased in COVID-19 and controls, respectively. Node size is proportional to the number of cells in each population, and the outer circle on each node denotes self-interactions. (**B**) Scatter plots showing the incoming and outgoing signaling activity of major immune populations in the healthy control group (upper panel) and the COVID-19 group (lower panel). Dot size represents the number of inferred ligand–receptor links. (**C**) Heatmaps showing incoming (upper panel) and outgoing (lower panel) signaling activity among immune populations in the COVID-19 group. Color intensity represents relative signaling strength. (**D**) Chord diagram illustrating the top 10 ligand–receptor pairs originating from NINJ1^+^ monocyte subsets in the COVID-19 group. Each sector on the left represents one ligand–receptor pair, labelled as signaling pathway | ligand–receptor; several pairs may therefore belong to the same pathway. Sector width is proportional to the communication probability summed across all receiver populations, and ribbons indicate the individual sender–receiver relationships. Saturated colors denote NINJ1^+^ sender populations; lightened colors denote populations acting only as receivers.

**Figure 4:**
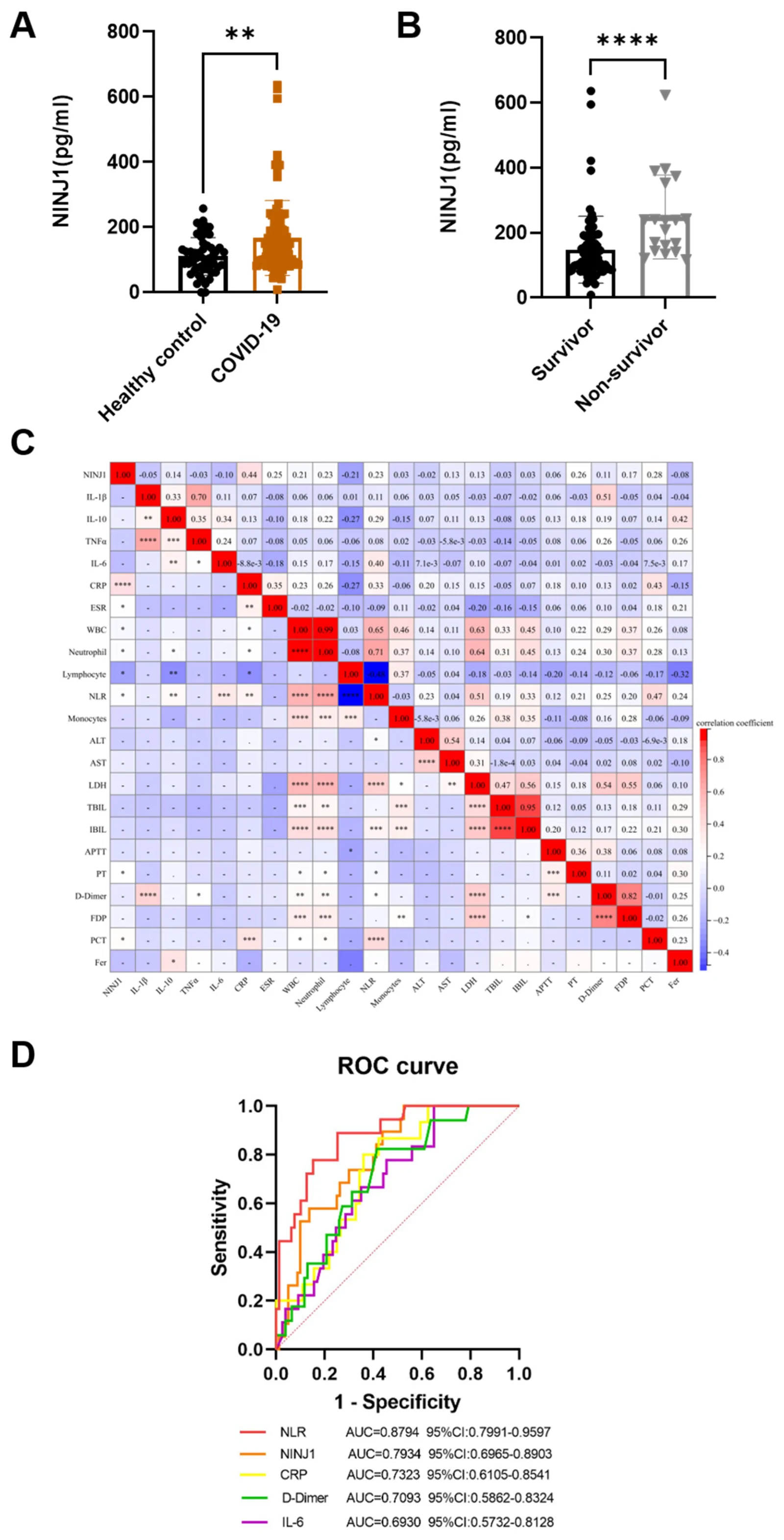
Circulating NINJ1 levels associated with inflammatory burden and mortality risk in COVID-19. (**A**) Serum NINJ1 concentrations in patients with COVID-19 and healthy controls. Statistical significance was determined using the Mann–Whitney U test. (**B**) Comparison of serum NINJ1 levels between COVID-19 survivors (n = 80) and non-survivors (n = 19). (**C**) Correlation heatmap showing associations between admission serum NINJ1 levels and clinical laboratory parameters, including C-reactive protein (CRP), procalcitonin (PCT), prothrombin time (PT), white blood cell count, neutrophil count, lymphocyte count, and neutrophil-to-lymphocyte ratio (NLR). Correlations were assessed using Spearman correlation analysis. (**D**) Receiver operating characteristic (ROC) curve analysis evaluating the predictive performance of NINJ1, NLR, CRP, D-dimer, and IL-6 for in-hospital mortality in COVID-19 patients. Area under the curve (AUC) values are shown for each biomarker. Statistical analyses were performed using the Mann–Whitney U test for comparisons between two independent groups. Correlation analyses were performed using Spearman’s rank correlation test. Data are presented as median with interquartile range (IQR). Statistical significance was defined as * *p* < 0.05; ** *p* < 0.01; *** *p* < 0.001; **** *p* < 0.0001 and - not significant.

**Table 1:** Performance Metrics for Mortality Prediction by Biomarkers.

Parameter	AUC (95%)	Cut-Off Value	SE%	SP%	Youden Index	*p* Value
NLR	0.8794 (0.7991–0.9597)	8.665	88.89	74.68	0.6357	<0.0001
NINJ1	0.7934 (0.6965–0.8903)	118.2	100	47.5	0.475	<0.0001
CRP	0.7323 (0.6105–0.8541)	52.05	86.67	57.81	0.4448	0.0053
D-D	0.7093 (0.5862–0.8324)	0.29	82.35	58.44	0.4079	0.0071
IL-6	0.6930 (0.5732–0.8128)	5.565	100	35.06	0.3506	0.0111

## Data Availability

The single-cell sequencing data (GSE149689) from public databases is available. Further details are available upon request from the corresponding author.

## Supplementary Materials

The following supporting information can be downloaded at https://www.mdpi.com/article/10.3390/biomedicines14102203/s1. Table S1: Patient Characteristics; Table S2: Patient Laboratory Test Results. Table S3: Donor identifiers and monocyte counts used in the SCENIC regulon analysis.
