# Supplementary material for: NINJ1 Defines a Hyperinflammatory Monocyte State Associated with Severe COVID-19
Source: Biomedicines. 2026 Sep 29;14(10):2203. doi: 10.3390/biomedicines14102203 (PMC13701203; doi:10.3390/biomedicines14102203)
Supplement: Supplementary file 1 [file biomedicines-14-02203-s001.zip › biomedicines-4511677-supplementary.pdf]

Supplementary Table S1. Patient Characteristics

	HC(n=51)	Survivor(n=80)	Non-survivor(n=19)
Male	20	52	12
Female	31	28	7
Age	38(45-53)	71（60-78.5）	86(83.75-89)

Supplementary Table S2. Patient Laboratory Test Results

	Survivor (n=80) Median (95% CI)	Non-survivor (n=19) Median (95% CI)	P-value
IL-1β (pg/mL）	5(5-8.22)	5.135(5-15.3)	0.146
IL-10 (pg/mL）	5.29(5-7.57)	10.75(7.203-22.75)	<0.01
TNFα (pg/mL）	13.4(9.27-16.6)	16.2(11.23-24.7)	0.042
IL-6 (pg/mL）	16.4(3.695-43.35)	41.35(16.88-85.15)	0.011
CRP (mg/L)	43.4(17.43-87.05)	80.6(65.2-106)	0.005
ESR (mm/h)	67(42-92)	60(37.5-89.85)	0.404
WBC(×10^9/L)	5.7(4.425-8.175)	12.75(7.375-17.33)	<0.01
Neutrophil(×10^9/L)	4.05(2.9-6.75)	11.9(6.075-16.23)	<0.01
Lymphocyte(×10^9/L)	0.8(0.5-1.3)	0.45(0.2-0.8)	0.007
NLR	5.75(3.14-9.33)	22.13(11.81-40.00)	<0.01
Monocytes(×10^9/L)	0.5(0.3-0.7)	0.4(0.275-0.625)	0.233
ALT(U/L)	25.4(14.1-45.08)	35.35(15.98-56.35)	0.394
AST(U/L)	28.9(20.08-46.6)	40.6(25.55-60.6)	0.134
LDH(U/L)	246.5(167.8-292.3)	404(257,593.5)	0.001
TBIL(μmol/L)	9.75(7.025-13.4)	9.85(7.15-15.38)	0.623
IBIL(μmol/L)	3.6(2.625-4.85)	4.4(3.275-6.625)	0.025
APTT(s)	27.5(25.9-30.4)	31.55(27.4-34.63)	0.018
PT(s)	11.7(11.25-12.73)	12.55(12.05-14.05)	0.01
D-dimer(mg/L)	0.25(0.13-0.69)	0.64(0.305-2.13)	0.007
FDP (mg/L)	2.5(1.75-6.55)	5.1(2.55-18.1)	0.024
PCT (ng/mL)	0.052(0.05-0.1458)	1.939(0.2205-5.11)	<0.01
Fer(ng/mL)	873(539-1147)	991.5(676.3-1141)	0.697

Note: non-normal distribution parameter data are expressed as median (25% and 75% interquartile range)

WBC: White blood cell

CRP: C-reactive protein

PCT: Procalcitonin

APTT: Activated partial thromboplastin time

PT: Prothrombin time

LDH:
ALT: Alanine aminotransferase
AST: Aspartate transaminase
TBIL: Total bilirubin

Supplementary Table S3. Donor identifiers and monocyte counts used in the SCENIC regulon analysis

Original sample ID	Donor ID in figure	Group	Monocytes (NINJ1+)	Monocytes (NINJ1-)
Sample 1	COVID-01	COVID-19	240	315
Sample 2	COVID-02	COVID-19	101	62
Sample 3	COVID-03	COVID-19	11	52
Sample 4	COVID-04	COVID-19	44	196
Sample 5	COVID-05	COVID-19	206	130
Sample 6	COVID-06	COVID-19	193	148
Sample 7	COVID-07	COVID-19	14	114
Sample 8	COVID-08	COVID-19	39	50
Sample 9	COVID-09	COVID-19	95	73
Sample 10	COVID-10	COVID-19	243	140
Sample 11	COVID-11	COVID-19	303	209
